# Comparing complications of Rezum and Urolift for BPH/LUTS using the Accordion Severity Grading System

**DOI:** 10.1007/s11255-025-04704-x

**Published:** 2025-07-29

**Authors:** Kevin McVary, Bronwyn Long, Amandip Cheema, Larry E. Miller

**Affiliations:** 1 Center for Male Health, Department of Urology, Stritch School of Medicine, Loyola University Medical Center, Maywood, IL USA; 2 Miller Scientific, 3101 Browns Mill Road, Ste 6, #311, Johnson City, TN 37604 USA

**Keywords:** Accordion, BPH, LUTS, Prostatic urethral lift, Rezum, Urolift

## Abstract

**Purpose:**

To compare the short- and long-term complication profiles of Rezum and Urolift using standardized approaches.

**Methods:**

We analyzed published data from the pivotal randomized trials of Rezum and Urolift. The severity of complications was independently graded by urologists using the Accordion Severity Grading System, developed by the American College of Surgeons National Surgical Quality Improvement Program. Short- (3 months) and long-term (5 years) complication rates were assessed. Long-term complications included medical retreatment, surgical retreatment, and surgical implant removal. We calculated the weighted postoperative morbidity index (PMI) values (0–1 scale) for each procedure and performed Monte Carlo simulations to account for uncertainty in complication rates and severities.

**Results:**

Short-term PMI values were similar between the Rezum (0.091) and Urolift (0.092) groups, with dysuria, hematuria, pain, and urinary urgency most commonly reported. Over 5 years, the cumulative complication rates were 15.4% for Rezum and 33.6% for Urolift. The associated 5-year PMI was 0.055 for Rezum and 0.165 for Urolift, indicating a three-fold higher long-term severity-weighted complication burden with Urolift. Monte Carlo simulations confirmed the robustness of these findings.

**Conclusions:**

This study identified significant differences in the long-term complication profiles of Rezum and Urolift when considering both the incidence and severity of postoperative complications. These findings may help guide clinical decision-making when selecting minimally invasive surgical options for BPH/LUTS treatment.

**Supplementary Information:**

The online version contains supplementary material available at 10.1007/s11255-025-04704-x.

## Introduction

Benign prostatic hyperplasia (BPH) is a prevalent condition affecting aging men, often manifesting as bothersome lower urinary tract symptoms (LUTS) that interfere with daily functioning and negatively impact quality of life [1]. The initial management of BPH/LUTS typically involves pharmacotherapy with alpha-blockers or 5-alpha reductase inhibitors. However, 30–60% of patients discontinue treatment within the first year due to adverse effects or inadequate symptom relief [2–4]. For patients who fail medical therapy or have contraindications to pharmacological management, transurethral resection of the prostate (TURP) has long been considered the standard surgical treatment [5, 6]. However, TURP is associated with significant complications such as bleeding, sexual dysfunction, and potential anesthetic risks, particularly in older individuals with comorbidities. These limitations have driven the development and adoption of minimally invasive surgical therapies (MISTs) aimed at providing comparable efficacy to TURP while reducing associated morbidity [7]. Recent analyses highlight both the expanding range of surgical techniques for BPH, from traditional approaches such as TURP to novel surgical treatments, and the evolving indications for intervention in contemporary practice, with patient selection increasingly individualized [8].

Two MISTs that have gained prominence are Rezum (Boston Scientific, Marlborough, MA, USA) and Urolift (NeoTract, Pleasanton, CA, USA). Rezum involves transurethral delivery of radiofrequency-generated water vapor to induce thermal necrosis and ablation of prostatic tissue [9]. In contrast, Urolift employs a mechanical approach involving the placement of permanent metal implants to retract obstructive prostatic lobes [10]. Although both procedures have demonstrated efficacy in alleviating BPH/LUTS [11], their distinct mechanisms of action may result in different complication profiles. Although studies of MISTSs often report the frequency and severity of complications, quantitative comparisons that integrate both frequency and severity are lacking, leaving a significant knowledge gap in guiding clinical decision-making when selecting between these options.

To address this gap, we used the Accordion Severity Grading System developed by the American College of Surgeons National Surgical Quality Improvement Program (ACS-NSQIP) [12] to compare the burden of complications using the postoperative morbidity index (PMI). The objective of this study was to quantify and compare the short-term (3 months) and long-term (5 years) complication profiles of each procedure.

## Methods

### Data sources

We analyzed published data from the multicenter randomized pivotal trials that evaluated the safety and efficacy of Rezum and Urolift in the treatment of BPH/LUTS. The treatment arms in the respective studies included 136 patients treated with Rezum [13] and 140 patients treated with Urolift [14]. These trials were selected on the basis of their status as pivotal studies for each procedure and their comparable inclusion criteria. Both trials enrolled men with prostate volumes of 30–80 cc, postvoid residual volume ≤ 250 ml, American Urological Association Symptom Score ≥ 13, and abnormal peak flow rates (≤ 15 ml/s for Rezum; ≤ 12 ml/s for Urolift).

### Complication Identification and Grading

We used the Accordion Severity Grading System to compare the complication profiles of Rezum and Urolift [12]. This system uses a 6-point scale to categorize postoperative complications. Grade 1 indicates a minor invasive procedure that can be performed at the bedside (e.g., urinary catheter), grade 2 indicates pharmacological treatment, grade 3 indicates an invasive procedure (e.g., reoperation or other endoscopic procedure) without general anesthesia, grade 4 indicates an operation under general anesthesia, grade 5 indicates organ system failure, and grade 6 indicates postoperative death. Although originally established to assess short-term morbidity, the scale has also been applied to evaluate long-term clinical outcomes across a range of surgical specialties [15–18].

Complications were categorized into short- (3 months) and long-term (5 years) outcomes. For short-term complications, we used a probabilistic approach based on the inclusion–exclusion principle [19] to estimate the proportion of patients experiencing at least one complication since only individual complication rates were reported in the studies. Long-term outcomes included medical retreatment, surgical retreatment, and surgical implant removal over 5 years. These variables were selected a priori because of their negative impact on patient outcomes and clear identifiability as distinct events within the reviewed manuscripts [20].

Two urologist authors (Blinded for peer review) independently graded each complication, and a third urologist (Blinded for peer review) adjudicated any disagreement. For complications potentially spanning multiple severity grades, the most representative grade was assigned with minimum and maximum plausible grades determined based on clinical experience.

### Postoperative morbidity index and complication severity analysis

We reported the incidence and severity of all short- and long-term complications. For long-term complications, we determined the 5-year cumulative incidence for each MIST. We also computed a weighted PMI for each MIST based on the incidence and severity of complications reported over 3 months and 5 years, respectively. The PMI calculation involved summing the severity weights of all complications experienced by patients undergoing each MIST and dividing them by the number of treated patients [21]. The PMI scale ranges from 0.0 to 1.0, where 0.0 indicates no postoperative complications and 1.0 indicates that every patient died postoperatively.

### Statistical analysis

We assessed the inter-rater reliability for complication grading using the intraclass correlation coefficient where values < 0.50 indicate poor reliability, 0.50–0.75 indicate moderate reliability, 0.75–0.90 indicate good reliability, and > 0.90 indicate excellent reliability [22]. We descriptively reported the incidence of individual complications, the cumulative incidence of long-term complications, and the PMI for each procedure. Monte Carlo simulations were performed to account for uncertainty in both complication rates and severities. Because complication rates were reported for each complication separately in the source studies, we used a triangular distribution bounded by the minimum and maximum possible rates to model short-term complication frequencies using the inclusion–exclusion principle [19]. We sampled long-term complication frequencies from binomial distributions based on the calculated 5-year event rates and 95% confidence intervals. For long-term complication grading, we employed a triangular distribution to simulate the grades for each complication, with the minimum, typical, and maximum values determined by independent grading from the urologists. The simulated probabilities and complication grade weights were multiplied to calculate the PMI distribution for each treatment. We generated 95% credible intervals for the PMI in each group through 1 000 iterations of these simulations. All statistical analyses were performed using Stata version 18.5 (StataCorp LLC, College Station, TX, USA).

## Results

**Fig. 1:**
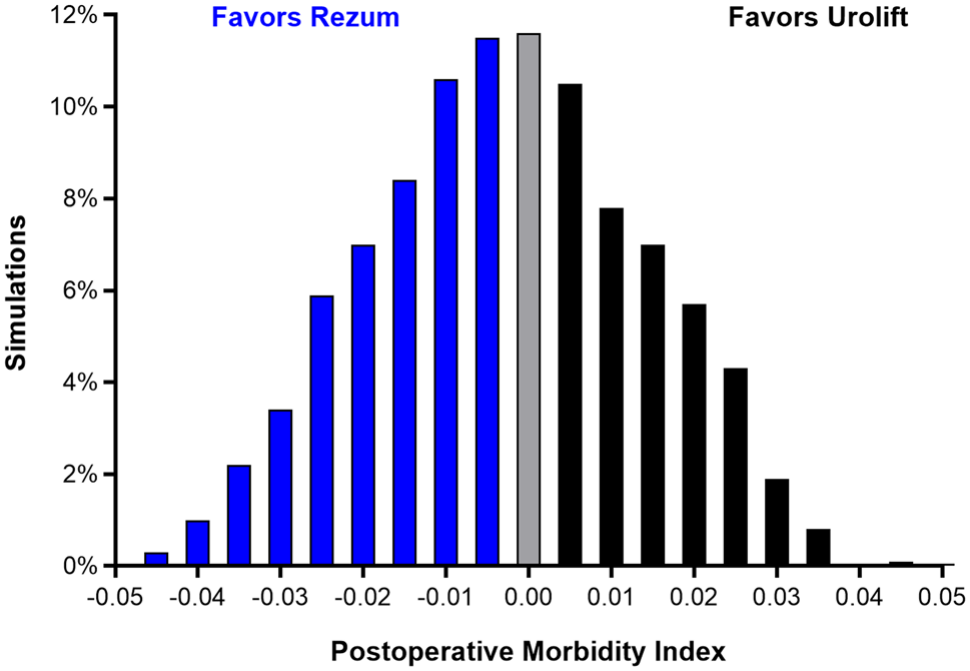
Distribution of short-term (≤ 3 months) Postoperative Morbidity Index (PMI) differences between Rezum and Urolift based on Monte Carlo simulations. The PMI through 3 months was 0.091 for Rezum and 0.092 for Urolift, with a difference between groups of −0.001 (95% credible interval: −0.033 to 0.022). A total of 56.9% of simulations favored Rezum, which demonstrates comparable overall short-term complication burden between the procedures

The inter-rater reliability for complication grading demonstrated excellent consistency, with an intraclass correlation of 0.96 across all complication grades. Through 3 months of follow-up, the most common mild or moderate (grade 1 or 2) complications were dysuria (Rezum 16.9%; Urolift 34.3%), hematuria (Rezum 11.8%; Urolift 25.7%), pain (Rezum 2.9%; Urolift 17.9%), and urinary urgency (Rezum 5.9%; Urolift 7.1%). Severe (grade 3 +) short-term complications were rare in both groups (Supplement Table 1). The overall short-term complication burden was comparable between Rezum and Urolift (PMI = 0.091 vs. 0.092). In Monte Carlo simulations, the difference in short-term PMI between Rezum and Urolift was negligible (−0.001), with 56.9% of simulations favoring Rezum (Fig. 1).

**Fig. 2:**
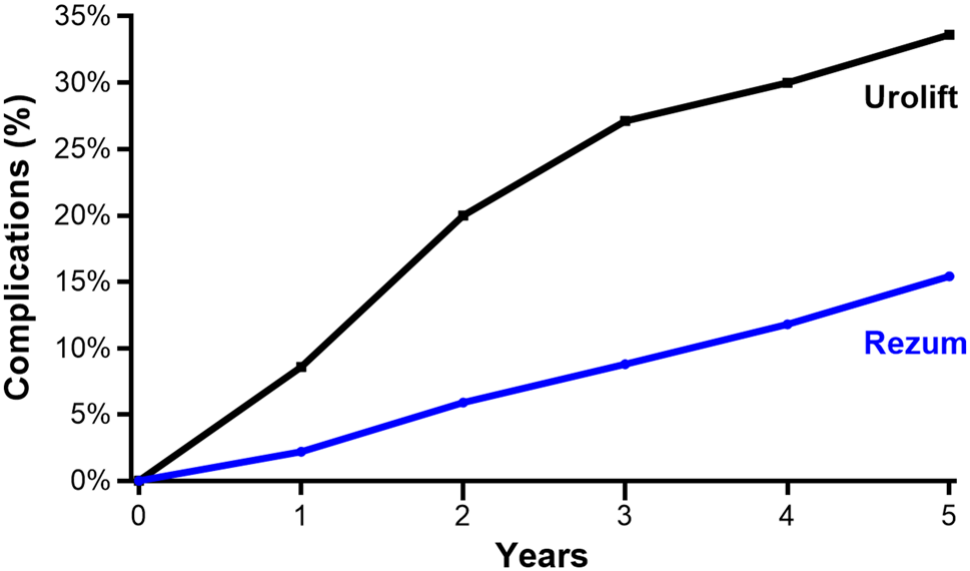
Cumulative complication rates for Rezum and Urolift over 5 years. At 5 years, rates were 15.4% with Rezum and 33.6% with Urolift, with a progressively widening gap in complication rates between the procedures

**Fig. 3:**
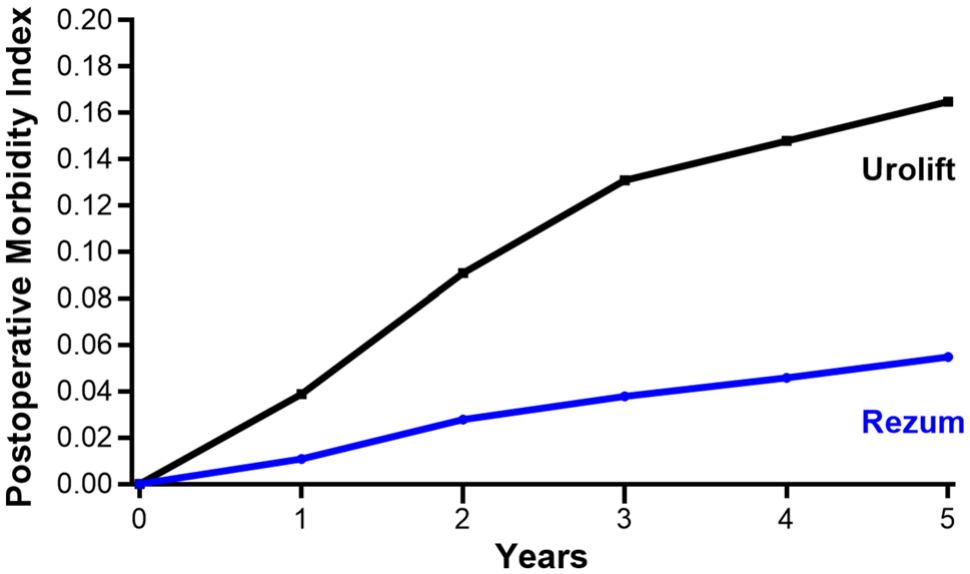
Annual weighted Postoperative Morbidity Index (PMI) for Rezum and Urolift over 5 years. At 5 years, the PMI was 0.055 with Rezum and 0.165 with Urolift, with a progressively widening gap in PMI between the procedures

**Fig. 4:**
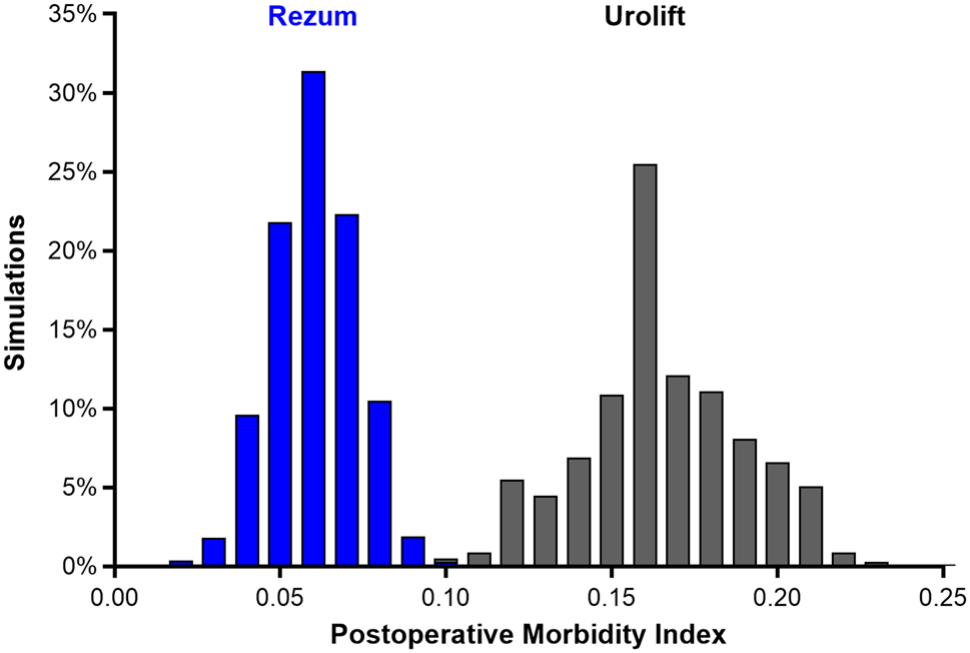
Distribution of 5-year Postoperative Morbidity Index (PMI) values for Rezum and Urolift based on Monte Carlo simulations. The PMI of each treatment over 5 years was 0.055 (95% credible interval: 0.027–0.086) for Rezum and 0.165 (95% credible interval: 0.110–0.224) for Urolift, with no overlap between groups

The most common long-term complication was medical retreatment (Grade 2), occurring in 11.0% of Rezum patients and 10.7% of Urolift patients. Surgical retreatment (Grade 4) was less frequent with Rezum than Urolift (4.4% vs. 13.6%). Surgical removal of metal implants (Grade 4), a complication specific to Urolift, was reported in 9.3% of these patients (Supplement Table 2). The cumulative complication rate throughout the 5-year follow-up period was consistently lower with Rezum than with Urolift. At 1 year, the cumulative rate was 2.2% in the Rezum group and 8.6% in the Urolift group. This difference increased over time, with 15.4% in the Rezum group and 33.6% in the Urolift group at 5 years (Fig. 2). The PMI over 5 years was 0.055 for Rezum and 0.165 for Urolift, indicating a three-fold higher long-term complication burden with Urolift (Fig. 3). Approximately half of the observed difference in long-term complication rates was attributable to surgical implant removal, which occurred exclusively in Urolift patients, with the remaining difference explained by the higher rate of surgical retreatment in the Urolift group. Monte Carlo simulations corroborated this finding, yielding non-overlapping 95% credible intervals for the PMI values, with Rezum demonstrating a lower complication burden in all 1000 simulations (Fig. 4).

## Discussion

The assessment and comparison of surgical complication profiles require accurate and standardized grading of postoperative complications. Although urological surgery studies typically report the frequency of complications, they often fail to fully characterize their severity. Our study addressed this research gap by utilizing the Accordion Severity Grading System to provide a standardized comparison of Rezum and Urolift accounting for both complication incidence and severity. To our knowledge, this is the first study to use PMI to quantitatively compare the complication profiles of MISTs for BPH/LUTS.

This study identified three key findings regarding Rezum and Urolift complication profiles. First, both procedures demonstrated favorably low short-term complication burdens, with most complications classified as mild (Grade 1). This suggests that both Rezum and Urolift are well-tolerated in the immediate postoperative period. Second, over a 5-year follow-up period, Urolift was associated with a substantially higher incidence of surgical reinterventions compared to Rezum (22.9% vs. 4.4%). Third, the long-term PMI was considerably higher with Urolift than Rezum (0.165 vs. 0.055), indicating a three-fold higher long-term complication burden for patients treated with Urolift.

The results of this study align with and expand upon previous studies reporting high surgical reintervention rates and low treatment durability rates with Urolift. A meta-analysis of Urolift studies reported a 30% surgical reintervention rate over 5 years [23], while a separate analysis that considered medical retreatment, surgical retreatment, and treatment effectiveness estimated the overall treatment durability of Urolift at just over 50% [24]. Our study extends these findings by applying, for the first time, a standardized complication grading system that indicates the postoperative morbidity burden is three-fold higher with Urolift than Rezum. The difference in postoperative morbidity burden was largely driven by higher rates of surgical retreatment and surgical removal of metal implants over 5 years in the Urolift group. While a meta-analysis reported that no specific patient characteristics were able to accurately predict retreatment risk following Urolift [23], improper implant placement, particularly when implants protrude into the bladder, increases the risk of encrustation and the need for implant removal [25]. Determination of the risk factors for retreatment with both therapies warrants further study.

These findings have important implications for physician–patient discussions regarding the choice of MIST for BPH/LUTS. The comparably low short-term complication burden of Rezum and Urolift may help alleviate patient concerns regarding immediate postoperative safety. However, the considerable differences in the long-term risk of surgical retreatment and associated higher PMI with Urolift warrant careful consideration. Physicians can use these results to provide a more comprehensive and balanced perspective of these treatments by emphasizing the potential trade-offs between short-term and long-term complication risks. This information may also enable patients to make informed decisions that align with their preferences and risk tolerances.

The PMI scores derived from this study may also inform postoperative follow-up protocols. Patients undergoing treatment with higher PMI scores may benefit from more intensive monitoring to promptly identify and manage complications. Conversely, procedures with lower PMI scores might allow for less frequent follow-up owing to the lower risk of severe complications. This risk-based approach to patient management could optimize healthcare resource utilization by reducing unnecessary clinic visits and diagnostic tests for lower-risk patients. Moreover, less frequent follow-up visits may reduce the patient burden by minimizing disruptions in daily life. This approach aligns with broader healthcare initiatives that support rational allocation of follow-up care based on evidence-driven determination of patient risk [26]. By modifying follow-up protocols based on procedure-specific PMI scores, healthcare providers can balance patient safety and improve postoperative care efficiency.

A key question emerging from these findings is whether LUTS/BPH should be viewed as a condition responsive to a single intervention or as a chronic disease that progresses over time, irrespective of treatment. The substantial difference in surgical reintervention rates between PUL and Rezum procedures suggests the limitations of PUL technology rather than the natural progression of LUTS/BPH. This distinction has important implications for healthcare quality metrics since clinicians may face Medicare Access and CHIP Reauthorization Act (MACRA) and Merit-based Incentive Payment System (MIPS) payment adjustments based on whether LUTS/BPH is evaluated by the acute surgical outcome or as a chronic condition requiring ongoing management [27]. This creates a pressing need for evidence to guide the appropriate classification of LUTS/BPH and the associated quality measures.

The methods used in this study offer several advantages over traditional complication reporting. First, it provides a more comprehensive understanding of the potential risks of these MISTs by considering both the incidence and severity of complications. Second, the use of a standardized grading system ensures consistency in evaluating complication severity across studies and facilitates comparisons between procedures. Third, Monte Carlo simulations improve the robustness of the findings by accounting for uncertainties in the source data.

Despite the strengths of this study, several limitations warrant further discussion. First, the analysis relies on published data from separate pivotal trials rather than individual patient data, requiring the estimation of aggregate complication incidence rates and severity grades. To partially mitigate this limitation, we identified unique patients for each long-term complication to enable accurate reporting of the 5-year data. Additionally, we used Monte Carlo simulations to account for potential variability in our estimates, which supports the primary findings. Second, while the analysis focused on long-term complications with the greatest clinical relevance to patients with BPH/LUTS, it may not capture all potential complications over the 5-year follow-up period. This could slightly underestimate the absolute PMI values for both procedures. However, given that this limitation applies equally to both Rezum and Urolift, it is unlikely to significantly alter the relative difference in the PMI between these two MISTs. Third, the assignment of severity grades based on clinical judgment introduced a degree of subjectivity. We mitigated this risk through independent physician assessments with high inter-rater agreement and Monte Carlo simulations to account for complication grade uncertainty. Fourth, while this study focused specifically on the complication burden of these procedures, we recognize that the choice of MIST should also consider factors such as treatment efficacy, erectile and ejaculatory function preservation, and patient preferences. Finally, the pivotal trials included relatively small samples and enrolled selected patients who may not reflect the broader population seen in real-world clinical practice, particularly with respect to age and comorbidities. Future research assessing outcomes in more diverse and representative patient groups would be valuable.

In conclusion, this study highlights significant differences in the long-term complication profiles of Rezum and Urolift when considering both the incidence and severity of postoperative complications. These findings may help guide clinical decision-making when selecting minimally invasive surgical options for BPH/LUTS treatment.

## Supplementary Information

Below is the link to the electronic supplementary material.

Supplementary file1 (DOCX 36 KB)

## Data Availability

Supporting data from this study will be made available upon submission of a data-sharing proposal specifying the project rationale, planned analyses, and intended use.
