# Supplementary material for: Comparing complications of Rezum and Urolift for BPH/LUTS using the Accordion Severity Grading System
Source: Int Urol Nephrol. 2025 Jul 29;58(2):545–50. doi: 10.1007/s11255-025-04704-x (PMC12864236; doi:10.1007/s11255-025-04704-x)
Supplement: Supplementary file 1 — Supplementary file1 (DOCX 36 KB) [file 11255_2025_4704_MOESM1_ESM.docx]

**SUPPLEMENTAL INFORMATION**

**Supplement Table 1. Incidence and Severity of Short-Term Complications (≤3 months) following Rezum and Urolift Procedures.**

| **Grade ^a^** | **Severity**  **Weight** | **Event** | | **Frequency (%)** | | | | |
| --- | --- | --- | --- | --- | --- | --- | --- | --- |
|  |  |  |  | **Urolift**  **(n=140)** | | **Rezum**  **(n=136)** | |  |
| **Mild** |  |  |  | |  | |  |  |
| 1 | 0.11 | Dysuria | | 34.3 | | 16.9 | |  |
|  |  | Hematuria | | 25.7 | | 11.8 | |  |
|  |  | Pain | | 17.9 | | 2.9 | |  |
|  |  | Urinary urgency | | 7.1 | | 5.9 | |  |
|  |  | Bladder spasm | | 3.6 | | 0.0 | |  |
|  |  | Ureteral catheterization/retention | | 0.7 | | 3.7 | |  |
|  |  | Clot retention (overnight stay) | | 0.7 | | 0.0 | |  |
|  |  | Hematospermia | | 0.0 | | 7.4 | |  |
|  |  | Ejaculatory volume decrease | | 0.0 | | 5.9 | |  |
|  |  | Urinary retention (extended) | | 0.0 | | 0.7 | |  |
|  |  | Nausea/vomiting (hospitalized) | | 0.0 | | 0.7 | |  |
| **Moderate** |  |  | |  | |  | |  |
| 2 | 0.26 | Urinary incontinence | | 3.6 | | 0.0 | |  |
|  |  | UTI | | 2.9 | | 7.4 | |  |
|  |  | Medical retreatment | | 0.7 | | 0.0 | |  |
|  |  | Urinary frequency | | 0.0 | | 5.9 | |  |
|  |  | Epididymitis | | 0.0 | | 2.9 | |  |
|  |  | New-onset ED | | 0.0 | | 0.0 | |  |
| **Severe** |  |  | |  | |  | |  |
| 3 | 0.37 | Urethral stricture | | 0.0 | | 0.0 | |  |
| 4 | 0.60 | Surgical retreatment | | 0.0 | | 0.7 | |  |
| 5 | 0.79 | Organ system failure | | 0.0 | | 0.0 | |  |
| **Death** |  |  | |  | |  | |  |
| 6 | 1.00 | Death | | 0.0 | | 0.0 | |  |

^a^Expanded Accordion Severity Classification of Postoperative Complications.

1=Requires only minor invasive procedures that can be done at the bedside such as insertion of intravenous lines, urinary catheters, and nasogastric tubes, and drainage of wound infections. Physiotherapy and the following drugs are allowed: antiemetics, antipyretics, analgesics, diuretics, and electrolytes.

2=Requires pharmacologic treatment with drugs other than such allowed for minor complications, for instance antibiotics. Blood transfusions and total parenteral nutrition are also included.

3=Invasive procedure without general anesthesia. Requires management by an endoscopic, interventional procedure or reoperation without general anesthesia.

4=Operation under general anesthesia. Requires management by an operation under general anesthesia.

5=Organ system failure.

6=Postoperative death.

**Supplement Table 2. Cumulative Rates of Long-Term Complications following Rezum and Urolift Procedures.**

| **Complication** | **Years** | | | | |
| --- | --- | --- | --- | --- | --- |
|  | **1** | **2** | **3** | **4** | **5** |
| Surgical retreatment | Rezum: 1.5  Urolift: 4.3 | Rezum: 3.7  Urolift: 7.1 | Rezum: 4.4  Urolift: 10.7 | Rezum: 4.4  Urolift: 13.6 | Rezum: 4.4  Urolift: 13.6 |
| Surgical implant removal | Rezum: ^a^  Urolift: 0.7 | Rezum: ^a^  Urolift: 4.3 | Rezum: ^a^  Urolift: 7.1 | Rezum: ^a^  Urolift: 7.1 | Rezum: ^a^  Urolift: 9.3 |
| Medical retreatment | Rezum: 0.7  Urolift: 3.6 | Rezum: 2.2  Urolift: 8.6 | Rezum: 4.4  Urolift: 9.3 | Rezum: 7.4  Urolift: 9.3 | Rezum: 11.0  Urolift: 10.7 |
| **TOTAL** | **Rezum: 2.2**  **Urolift: 8.6** | **Rezum: 5.9**  **Urolift: 20.0** | **Rezum: 8.8**  **Urolift: 27.1** | **Rezum: 11.8**  **Urolift: 30.0** | **Rezum: 15.4**  **Urolift: 33.6** |

^a^Not applicable.
